# Supplementary material for: Cultivar Differentiation and Origin Tracing of Panax quinquefolius Using Machine Learning Model-DrivenComparative Metabolomics
Source: Foods. 2025 Apr 14;14(8):1340. doi: 10.3390/foods14081340 (PMC12027468; doi:10.3390/foods14081340)
Supplement: Supplementary file 1 [file foods-14-01340-s001.zip › foods-3300227-supplementary.pdf]

Supplementary materials

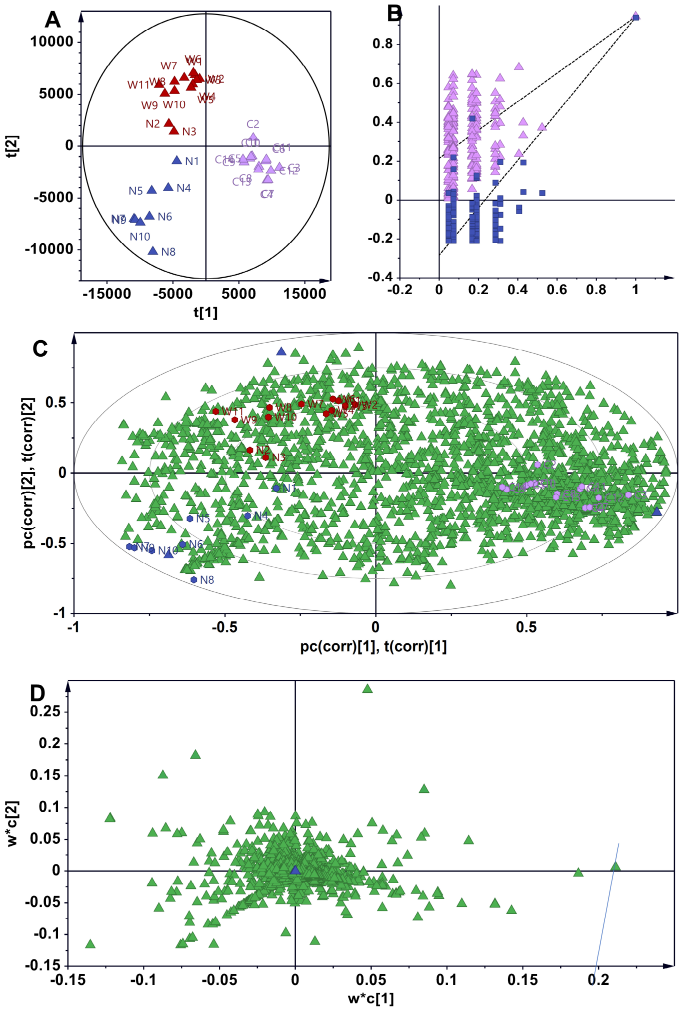

Sup Figure 1

Partial least squares-discriminant analysis for the differentiation of wild and cultivated American ginseng. (A) Partial Least Squares-Discriminant Analysis (PLS-DA) showed that the three groups of samples were separated from each other and clustered within the groups, and the samples of North American cultivated American ginseng group were in the second quadrant, the samples of Chinese ginseng group were in the second quadrant, and the samples of wild American ginseng group were in the fourth quadrant, which indicated that the wild American ginseng could be differentiated from the cultivated American ginseng. (B) The 200 permutation test indicates that the PLS-DA model is robust and reliable. (C) The differential metabolites with significant content fluctuations are at the outer edges around the scatter plot.

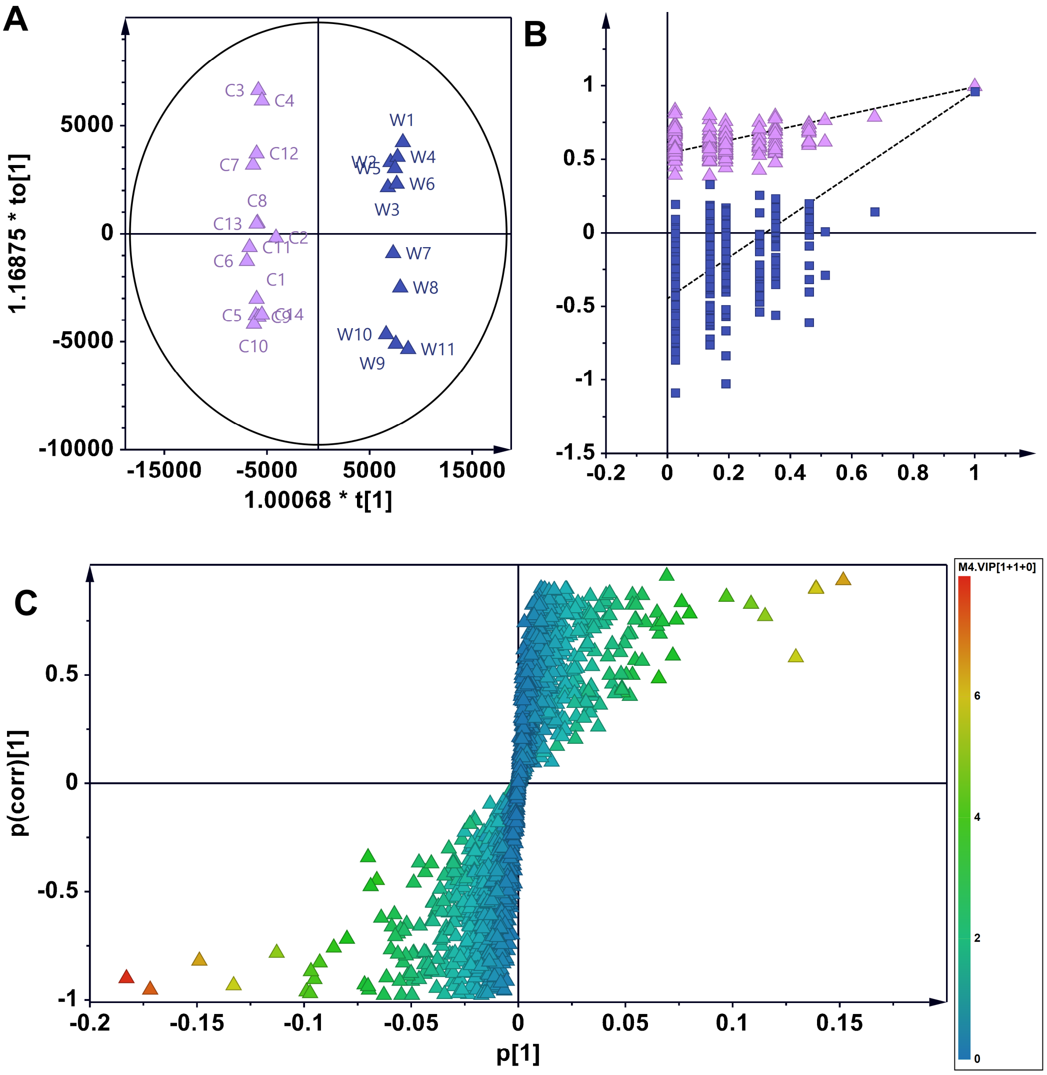

Sup Figure 2

Orthogonal partial least squares-discriminate analysis (OPLS-DA) screening of differential metabolites in wild American ginseng (C) and Chinese cultivated American ginseng (N) samples. (A) Orthogonal partial least squares-discriminate analysis (PLS-DA) showed intergroup separation and intragroup aggregation of wild and Chinese cultivated American ginseng samples. (B) The 200 permutation test indicates that the OPLS-DA model is robust and reliable. The compounds with significant content fluctuations were at the outer edges around the scoring plot. (C) Based on the screening criteria (VIP higher than 1 and absolute value of pcorr higher than 0.5), there were 243 differential metabolites screened. After preliminary identification 44 were annotated.

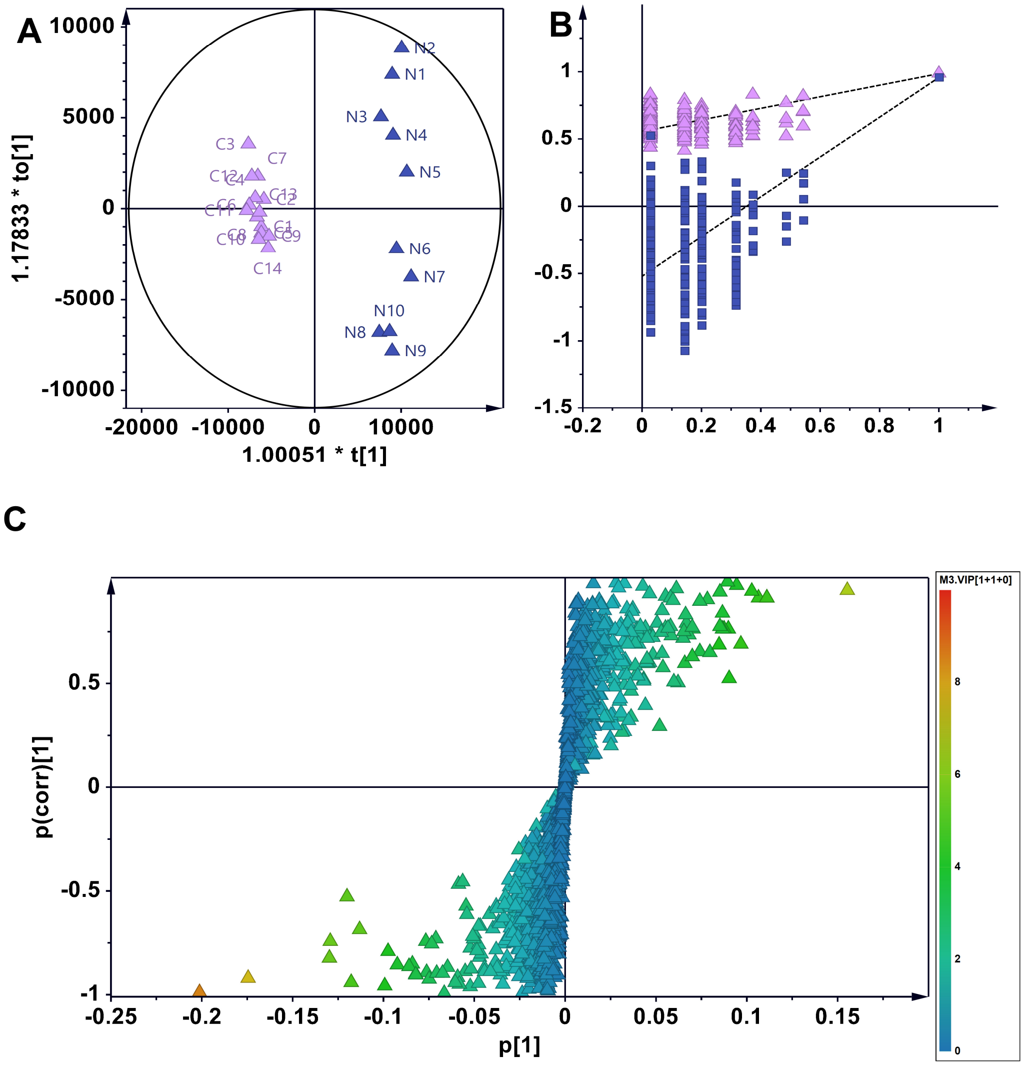

Sup Figure 3

OPLS-DA model showed the differential metabolites of North American cultivated American ginseng (W) and Chinese cultivated American ginseng (N) samples. (A) OPLS-DA analysis of intergroup separation and intragroup clustering. (B) The 200 permutation test indicates that the OPLS-DA model is robust and reliable. The compounds with significant content fluctuations are at the outer edges of the perimeter of the scoring plot. (C) Differential metabolite compounds with significant content fluctuations are at the periphery of the scoring plot.

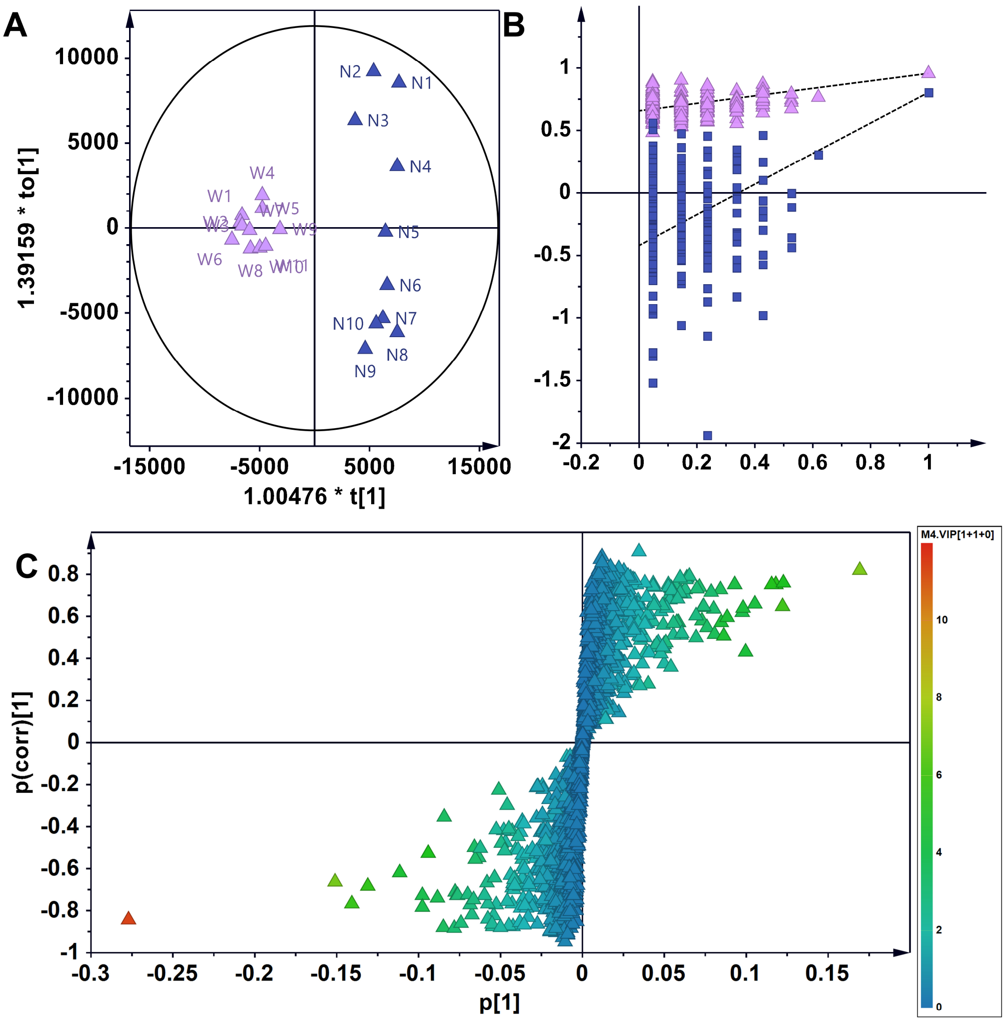

Sup Figure 4

OPLS-DA model showed the differential metabolites between wild American ginseng (C) and North American cultivated American ginseng (W) samples. (A) OPLS-DA analysis of intergroup separation and intragroup clustering. (B) The 200 permutation test indicates that the OPLS-DA model is robust and reliable. The compounds with significant content fluctuations are at the outer edges of the perimeter of the scoring plot. (C) Differential metabolite compounds with significant content fluctuations are at the periphery of the scoring plot. Thirty-eight differential metabolites were screened between wild American ginseng (C) and North American cultivated American ginseng (W), of which 24 differential metabolites were higher in North American cultivated American ginseng (W) and 14 were higher in wild American ginseng (C).
